# Genome-wide identification of conserved and novel microRNAs in one bud and two tender leaves of tea plant (*Camellia sinensis*) by small RNA sequencing, microarray-based hybridization and genome survey scaffold sequences

**DOI:** 10.1186/s12870-017-1169-1

**Published:** 2017-11-21

**Authors:** Anburaj Jeyaraj, Xiao Zhang, Yan Hou, Mingzhu Shangguan, Prabu Gajjeraman, Yeyun Li, Chaoling Wei

**Affiliations:** 1 State Key Laboratory of Tea Plant Biology and Utilization, Anhui Agricultural University, 130 Changjiang West Road, Hefei, Anhui Province 230036 People’s Republic of China; 2 Department of Biotechnology, Karpagam University, Coimbatore, India

**Keywords:** *Camellia sinensis*, MicroRNAs, Small RNA sequencing, Genome survey, miRNA microarray, Secondary metabolism

## Abstract

**Background:**

MicroRNAs (miRNAs) are important for plant growth and responses to environmental stresses via post-transcriptional regulation of gene expression. Tea, which is primarily produced from one bud and two tender leaves of the tea plant (*Camellia sinensis*), is one of the most popular non-alcoholic beverages worldwide owing to its abundance of secondary metabolites. A large number of miRNAs have been identified in various plants, including non-model species. However, due to the lack of reference genome sequences and/or information of tea plant genome survey scaffold sequences, discovery of miRNAs has been limited in *C. sinensis*.

**Results:**

Using small RNA sequencing, combined with our recently obtained genome survey data, we have identified and analyzed 175 conserved and 83 novel miRNAs mainly in one bud and two tender leaves of the tea plant. Among these, 93 conserved and 18 novel miRNAs were validated using miRNA microarray hybridization. In addition, the expression pattern of 11 conserved and 8 novel miRNAs were validated by stem-loop-qRT-PCR. A total of 716 potential target genes of identified miRNAs were predicted. Further, Gene Ontology (GO) and the Kyoto Encyclopedia of Genes and Genomes (KEGG) pathway analysis revealed that most of the target genes were primarily involved in stress response and enzymes related to phenylpropanoid biosynthesis. The predicted targets of 4 conserved miRNAs were further validated by 5’RLM-RACE. A negative correlation between expression profiles of 3 out of 4 conserved miRNAs (csn-miR160a-5p, csn-miR164a, csn-miR828 and csn-miR858a) and their targets (ARF17, NAC100, WER and MYB12 transcription factor) were observed.

**Conclusion:**

In summary, the present study is one of few such studies on miRNA detection and identification in the tea plant. The predicted target genes of majority of miRNAs encoded enzymes, transcription factors, and functional proteins. The miRNA–target transcription factor gene interactions may provide important clues about the regulatory mechanism of these miRNAs in the tea plant. The data reported in this study will make a huge contribution to knowledge on the potential miRNA regulators of the secondary metabolism pathway and other important biological processes in *C. sinensis*.

**Electronic supplementary material:**

The online version of this article (10.1186/s12870-017-1169-1) contains supplementary material, which is available to authorized users.

## Background

In plants, miRNAs negatively regulate gene expression at the post-transcriptional level by translational repression or degradation and silencing of target gene transcripts [1, 2]. Since the first miRNA (*lin 4*) was discovered in *Caenorhabditis elegans* [3], a large number of miRNAs have been identified across various species. To date, a total of 28,645 hairpin precursor miRNAs and 35,828 mature miRNAs have been deposited in public databases. Of these miRNAs, 6992 precursor miRNAs and 8496 mature miRNAs are found in various plant species (miRBase, Release 21), but none of these miRNAs are from plants of the *Theaceae* family (www.mirbase.org) [4]. Recent evidence has demonstrated that plant miRNAs are extensively involved in a number of biological functions including growth, development, and defense response against stresses [1, 5]. In light of this evidence, it would be important to identify the miRNAs present in this family of plants.

The tea plant [*Camellia sinensis* (L.) O. Kuntze], which belongs to the family *Theaceae*, originated in China. Usually, tea is processed from one bud and the two uppermost leaves on the tender shoots of tea plants. It is one of the most popular non-alcoholic beverages in the world because of its attractive aroma, taste, and health-promoting effects, which are attributable to the abundance of secondary metabolites present in tea plant leaves, including polyphenols, theanine, and volatile compounds [6]. Recently, a few studies have reported the miRNAs in *C. sinensis*. In the reported studies, several miRNAs in tea plants were detected by the comparative genomics approach [7–9] and direct cloning approach [10, 11]. However, these have provided relatively little information in validating these miRNAs and their functions in the tea plants.

Recent developments in next-generation high-throughput sequencing (HTS) technology have allowed researchers to identify novel and low-abundant miRNAs in non-model plant species [12]. In particular, genome survey sequences have better potential for predicting the pre-miRNA secondary structures than the expressed sequence tags (ESTs) of plant species whose genome sequences are not yet available in public databases [13–15]. Despite these advances in technology, very few studies have applied them to the investigation of miRNAs present in the tea plant. Recently, 106 conserved and 98 candidate novel miRNAs from tea were identified by HTS [16]. Similarly, Zheng et al. [17] reported 295 conserved and 72 potential novel miRNAs in tea plants by applying HTS. However, our knowledge of miRNAs present in *C. sinensis* is still limited due to the lack of whole-genome sequence information.

In this study, a small RNA library created from one bud and two tender leaves of tea shoots was constructed, and HTS was performed to obtain the miRNA profiles of *C. sinensis*. The potential secondary structures of the identified miRNAs were elucidated using the draft scaffold sequence assemblies of the *C. sinensis* genome that were obtained by genome survey using whole genome shotgun (WGS) sequencing. The expression profiles of the identified miRNAs were validated by miRNA microarrays, as well as stem-loop qRT-PCR. Further, the functions of the predicted potential miRNA targets were predicted using Gene Ontology (GO) and the Kyoto Encyclopedia of Genes and Genomes (KEGG) pathway. The target transcription factor genes of 4 conserved csn-miRNAs were validated through 5’RLM-RACE and correlation in the expression pattern of miRNAs and their target transcription factor genes were determined by qRT-PCR. The results of this study not only enrich the miRNA database of *Theaceae*, but also provide insights into the mechanism underlying posttranscriptional gene regulation mediated by miRNAs and lay the groundwork for further exploration of their biological roles in *C. sinensis* and other closely related species.

## Results

### Small RNA library construction and sequence analysis

**Fig. 1:**
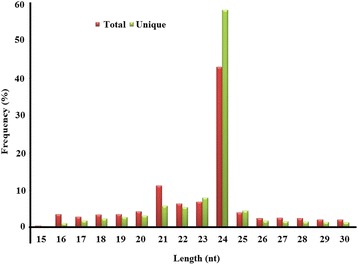
Size distribution and abundance of small RNAs obtained from HTS analysis

To identify the miRNAs in the tea plant, a small RNA library from the sample (one bud and two tender leaves) was constructed and subjected to HTS. A total of 6,211,111 raw reads were generated. After low-quality reads, adapters and short RNAs less than 15 nucleotides in length were removed, 3,455,797 unique small RNA reads (55.64% of the total raw reads) were obtained. These small RNA sequences were compared with the sequences deposited in the Repbase and Rfam databases, and an additional 35,464 reads representing rRNA, tRNA, snoRNA, snRNA and other non-coding RNAs were excluded. The sequences of the total and unique reads ranging from 15 to 30 nucleotides in length are shown in Fig. 1. The majority of small RNAs were 21–24 nucleotides in length, and 24-nucleotide small RNAs were predominant. The 24-nucleotide small RNAs comprised 43.0% and 58.29% of the total and unique sequences, respectively, and 21-nucleotide small RNAs comprised 11.16% and 5.76% of the total and unique sequences, respectively (Fig. 1). Sequences longer than 25 nucleotides and shorter than 17 nucleotides were discarded. The remaining 287,525 reads were retained for miRNA validation and prediction.

### Genome survey of the tea plant

We obtained 115.7 G base pairs (Gbp) of high-quality sequencing data from three DNA libraries (180 bp, 500 bp and 800 bp). The peak frequency of 17-mers in the read set was about 21 based on the 17-mer analysis of Li et al. [18]. The total k-mer count was 67,780,201,950. The tea genome size was estimated to be 3.22 Gb using the following formula: genome size = k-mer count/peak of the k-mer distribution. Thus, the genome survey data represented 27.2× coverage of *C. sinensis* genome. A total of 2,603,467 contigs were assembled with a total sequence length of 953.4 Mb. The N50 length was 668 bp in our assembly, and the longest contig and scaffold were 27,766 and 55,407 bp long respectively.

### Identification of conserved miRNAs

**Fig. 2:**
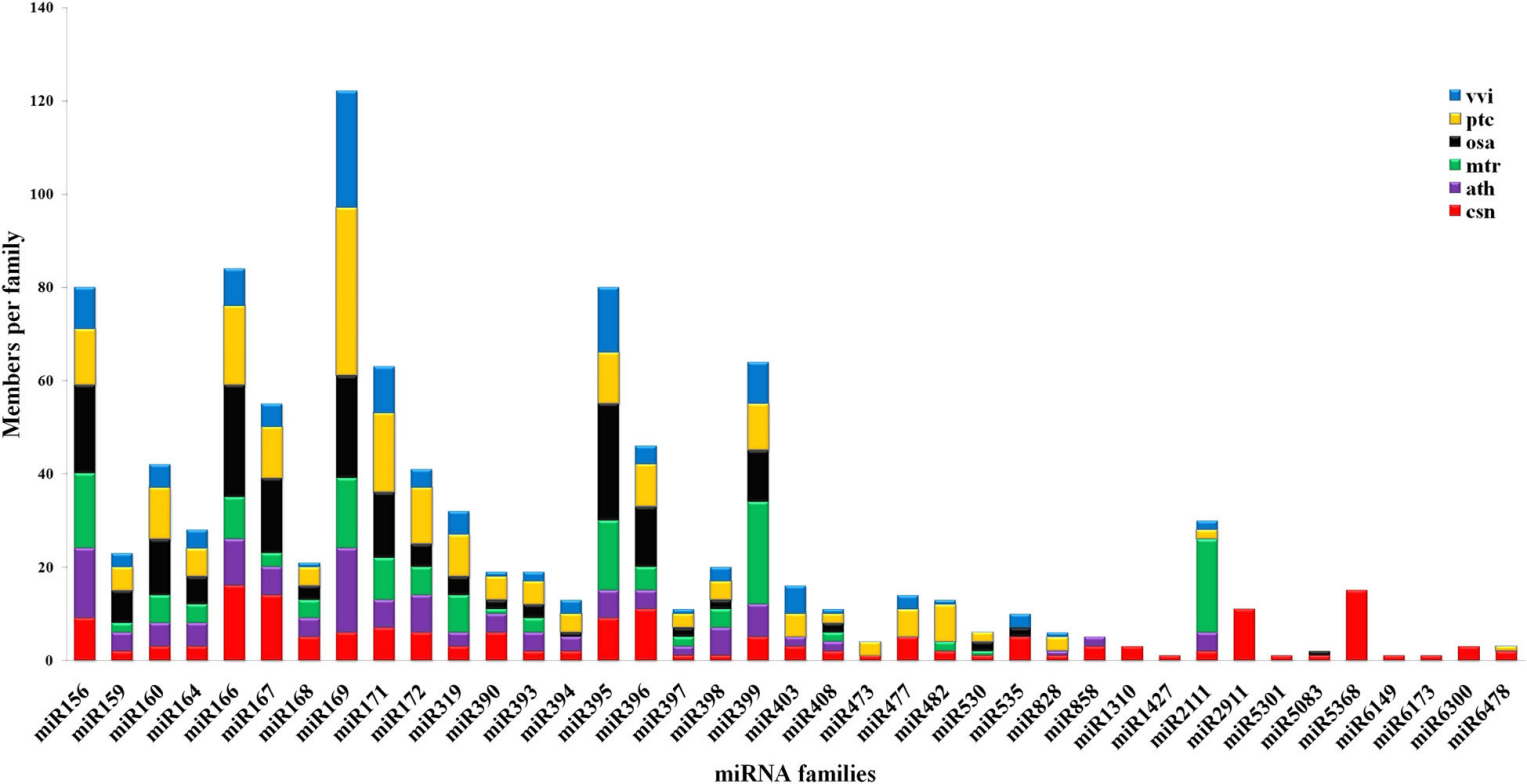
Number of paralogs per conserved miRNA family in six plant species: *Camellia sinensis* (*red*), *Populus trichocarpa* (*orange*), *Oryza sativa* (*black*), *Vitis vinifera* (*blue*), *Arabidopsis thaliana* (*purple*) and *Medicago truncatula* (*green*). The number of members of the miRNA family in the five plant species from miRBase and in *Camellia sinensis* obtained in the present study are listed

In order to identify the conserved miRNAs in *C. sinensis*, we compared the small RNA reads to mature and precursor miRNAs from other plant species deposited in miRBase 21 (http://www.mirbase.org/) based on the presence of the homologous “seed” regions with 0–3 mismatched bases in the mature region of known miRNAs [19]. Among the unique small RNA reads analyzed, 124,826 reads were mapped to known plant-specific miRNAs and represented a total of 175 miRNAs belonging to 39 conserved miRNA families across a variety of plant species (Additional file 1). We counted the number of members in the 39 conserved miRNA families and found that this number varied widely from 1 to 16 for each family. Among these families, the csn-miR166 family contained the highest number of individual miRNA members, with 16 members distinguished based on nucleotide differences. Csn-miR5368 (15 members) was followed by csn-miR167 (14 members); csn-miR396 and csn-miR2911 (11 members each); csn-miR156 and csn-miR395 (9 members each); csn-miR171 (7 members); and csn-miR169, csn-miR172 and csn-miR390 (6 members each). The remaining miRNA families comprised 1 to 5 members each (Fig. 2).

The read number of the conserved miRNAs varied greatly; thus, these miRNAs differed widely in their expression levels (Additional file 1). Among the identified 39 conserved miRNA families, csn-miR396 showed high expression with a total number of 59,922 reads (48% of the total conserved miRNA reads); it was followed by csn-miR166 (25,026 reads, 20.05%) and csn-miR159 (11,904 reads, 9.54%) (Additional file 1). In addition, the percentage distribution of reads for individual members within each family showed wide variations (Additional file 1).

To investigate the evolutionarily conserved nature of these conserved miRNAs, we compared each miRNA family member of *C. sinensis* against the miRNA sequences available in miRBase for *Populus trichocarpa, Medicago truncatula, Vitis vinifera, Oryza sativa* and *Arabidopsis thaliana* (Fig. 2). The results indicated that the miRNA families identified were present in related plant species; thus, their functions may be evolutionarily conserved in the selected plant species.

### Identification of novel miRNAs

The non-conserved small RNA reads were mapped to ESTs and scaffold sequences that were assembled at the base of the genome survey dataset (Additional file 2). The stem-loop structure of miRNA precursors was used to predict novel miRNAs using the mfold program [20], and 83 novel miRNAs were identified in the tea plant (Additional file 3).

All miRNA precursors had a standard stem-loop hairpin secondary structure (SS). These miRNA precursors had folding free energies ranging from −4.7 to −138.3 kcal/mol (average, −57.08 kcal/mol). The predicted precursors of these novel miRNAs were 38–258 nucleotides in length. The sequence is most likely to represent an miRNA when the minimal folding free energy index (MFEI) is more than 0.85 [21]. It was found that the MFEI values of these miRNAs ranged from 0.4 to 2.8, with most MFEIs being >0.85.

### qRT-PCR validation of the selected miRNAs

**Fig. 3:**
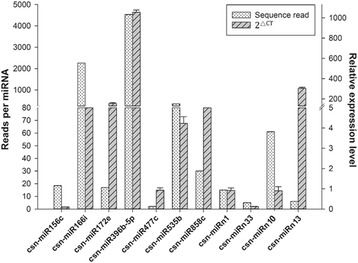
Relationship between the relative expression levels and HTS read counts of the identified csn-miRNAs. The expression level of U6 snRNA was used as an internal control. Relative expression was calculated using the 2^△CT^ method with stem-loop qRT-PCR. Data represent the mean ± SD values of three biological replicates

To validate the reliability of the miRNAs predicted using HTS, qRT-PCR was used to analyze seven conserved miRNAs (csn-miR156c, csn-miR166i, csn-miR172e, csn-miR396b-5p, csn-miR477c, csn-miR535b and csn-miR858c) and four novel miRNAs (csn-miRn1, csn-miRn10, csn-miRn13 and csn-miRn33), which represented different expression levels with read counts ranging from 3 to 4636. The qRT-PCR results were highly consistent with the small RNA sequencing data (Fig. 3).

**Fig. 4:**
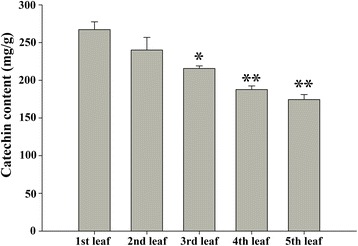
The contents of catechin in leaf tissues from different positions in the tender tea shoot. Data represent the means ± SD (*n* = 3) of three biological samples. * denotes the significance levels. * *p* < 0.05; ** *p* < 0.001

**Fig. 5:**
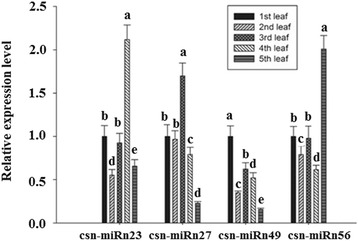
Relative expression levels of four selected novel csn-miRNAs in leaf tissues from different positions in the tender tea shoot. U6 snRNA was used as an internal control. The expression level of the miRNAs in the first leaves was set as 1.0. Relative expression was calculated using the 2^-△△CT^ method with stem-loop qRT-PCR. Data represent the mean ± SD values of three biological replicates. Different letters above the bars represent significant differences at *p* < 0.05. Means followed by the same letter over the bars are not significantly different at the 0.5% level, according to DMRT analysis

To further validate the authenticity of the novel miRNAs and gain insight into their potential functions, the expression profiles of four predicted miRNAs (csn-miRn23, csn-miRn27, csn-miRn49 and csn-miRn56) in the leaves at different positions on the tender tea shoots were investigated. In addition, we also measured catechin contents in the corresponding leaves. The content of catechin was significantly higher in the 1st leaf, followed by 2nd leaf, 3rd leaf, 4th leaf and 5th leaf. These results indicated that the level of catechin was gradually decreased from 1st leaf to 5th leaf (Fig. 4). The expression pattern of csn-miRn23 was higher in 4th leaf, followed by 3rd leaf and 2nd leaf, with less expression detected in 5th leaf in comparison to 1st leaf. This expression pattern suggests that csn-miRn23 is negatively correlated with the pattern of catechin content. The expression pattern of csn-miRn49 was dramatically fluctuated in the leaves at different positions on the tender tea shoots: the lowest expression was observed in 5th leaf, followed by 2nd leaf, 4th leaf and 3rd leaf. Csn-miRn27 and csn-miRn56 showed no obvious correlation between expression and catechin content: the highest expression pattern of csn-miRn27 and csn-miRn56 were observed in 3rd leaf and 5th leaf while the lowest expressions were observed in 5th leaf and 4th leaf respectively (Fig. 5). It requires further investigations to understand the relationship between catechin contents and these identified novel miRNAs in the leaves of tea plant.

### Microarray analysis of miRNAs

Microarray-based hybridization was employed to confirm the existence of conserved and novel miRNAs predicted in this study. The mixed RNA pool microarray consisted of 258 probes that represented all the predicted miRNAs from HTS. The small-molecular-weight RNAs isolated from one bud and two tender leaves were hybridized to the microarray chip. A total of 111 miRNAs were detected by microarray analysis, of which 93 were conserved miRNAs and 18 were novel miRNAs (Additional file 4). The conserved miRNA family members of csn-miR5368, csn-miR6173, csn-miR2911, and csn-miR6300 displayed high levels of expression, whereas those beloning to the csn-miR477c, csn-miR482-5p, csn-miR858b, csn-miR156, csn-miR395 and csn-miR403 families showed low levels of expression. With regard to the novel csn-miRNAs, csn-miRn5 and csn-miRn11-3p showed higher expression signals than the other putative novel miRNAs (Additional file 4).

### Prediction of miRNA target genes

To help elucidate the biological functions of the identified miRNAs, we searched for the complimentary mRNA sequences from the corresponding transcriptome sequence data of *C. sinensis* to predict potential targets of the miRNAs using the Target Finder program. A total of 716 potential target genes were identified for 187 miRNAs, including 116 conserved and 71 novel miRNAs, based on their perfect or near-perfect complementarity to their target mRNA sequences. For some miRNAs, more than one potential target gene was predicted. Detailed annotations of the results are presented in Additional file 5. Most of the conserved miRNA families were predicted to target transcription factor genes; this suggests that they may play a role in post-transcriptional regulation and transcriptional networks. Other miRNAs were predicted to target genes involved in diverse physiological and metabolic processes, including the regulation of plant metabolism, transport, cell growth and maintenance, and stress responses (Additional file 5).

### GO and KEGG analysis

**Fig. 6:**
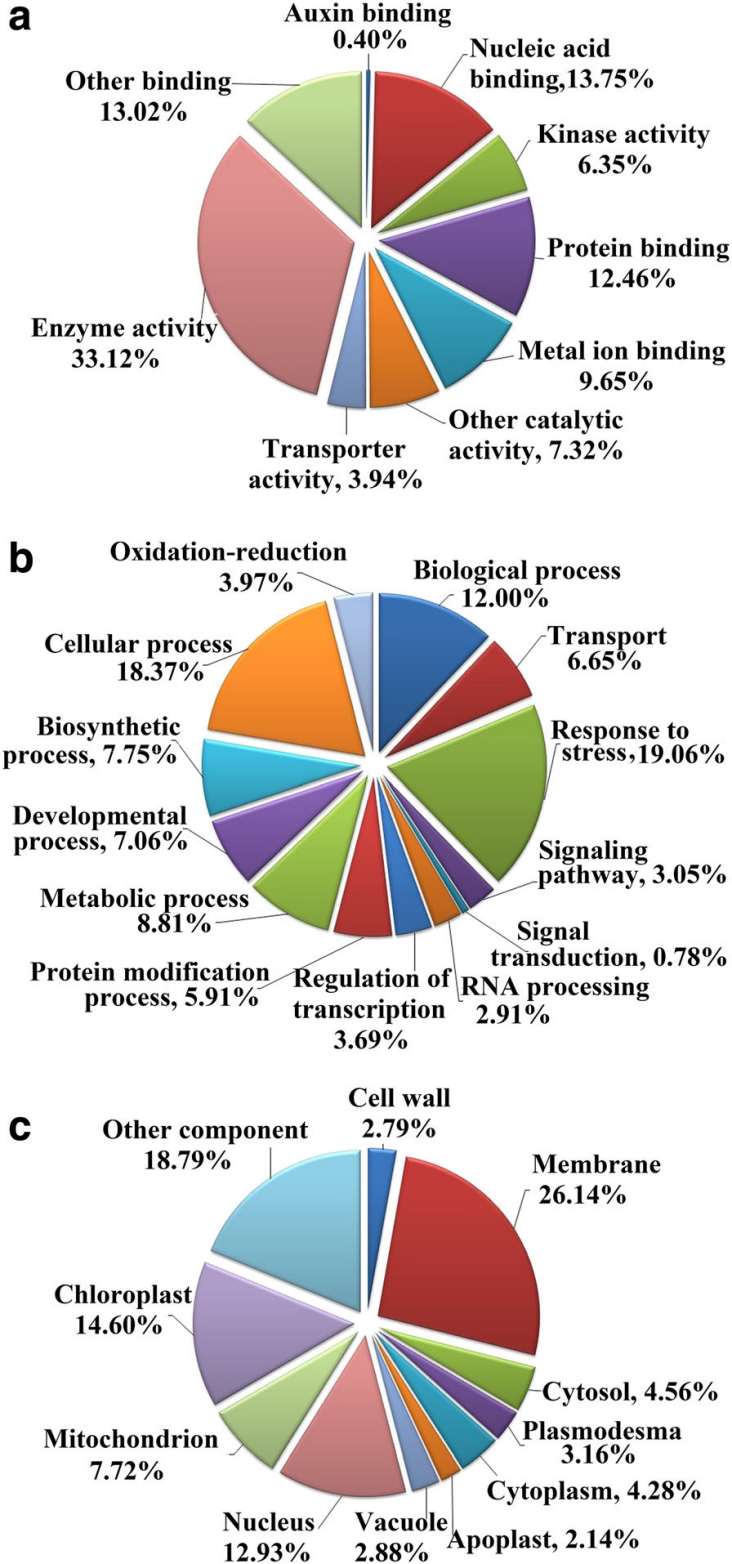
GO analysis of predicted putative target transcripts of csn-miRNAs. Categorization of csn-miRNA-target transcripts was performed according to molecular function (**a**), biological process (**b**) and cellular component (**c**)

GO analysis of the predicted target transcripts of miRNAs was performed to understand their potential regulation in the tea plant [22]. Based on their functional annotations, the target genes were classified into three GO categories: molecular function, biological process and cellular component (Fig. 6 and Additional file 6). Molecular function was represnted by 9 terms (Fig. 6a), with the most frequent term being enzyme activity (33.12%); it was followed by nucleic acid binding (13.75%) and other binding (13.02%). Biological process was represented by 13 terms, with the three most frequent terms being response to stress (19.06%), cellular process (18.37%) and biological process (12%) (Fig. 6b). Most of the proteins encoded by the target transcripts were localized in the membrane (26.14%), followed by other cellular components (18.79%) and the chloroplast (14.60%) (Fig. 6c).

**Fig. 7:**
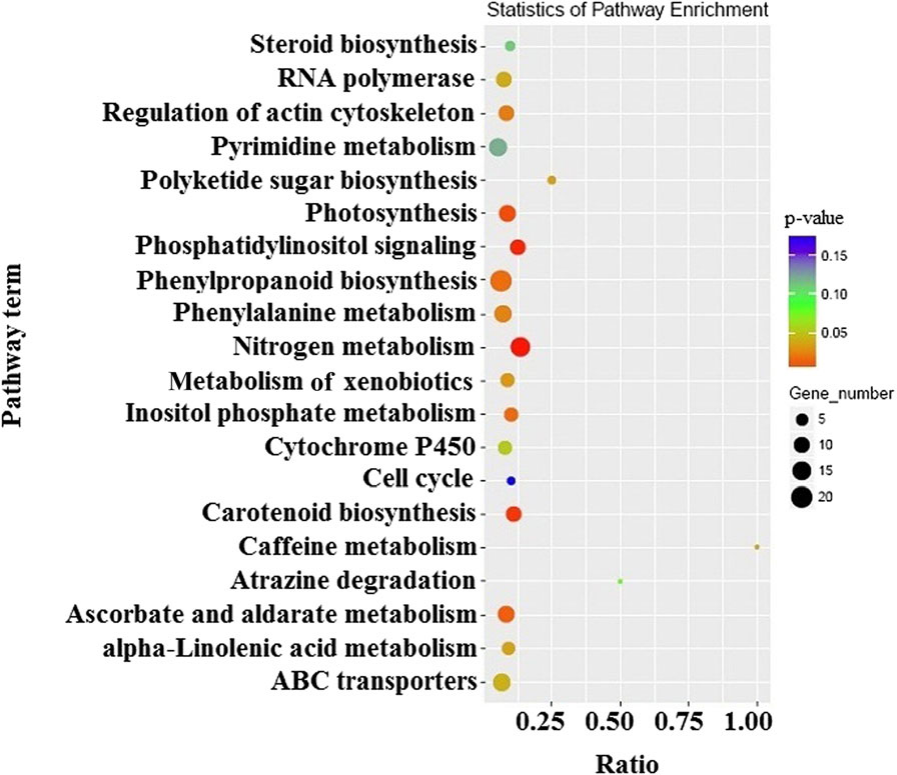
KEGG analysis of pathway enrichment for predicted putative target transcripts of miRNAs in the tea plant. The significance of the matched gene ratio represented by the coloring of the q-values; the circle size is proportional to the target gene number

**Fig. 8:**
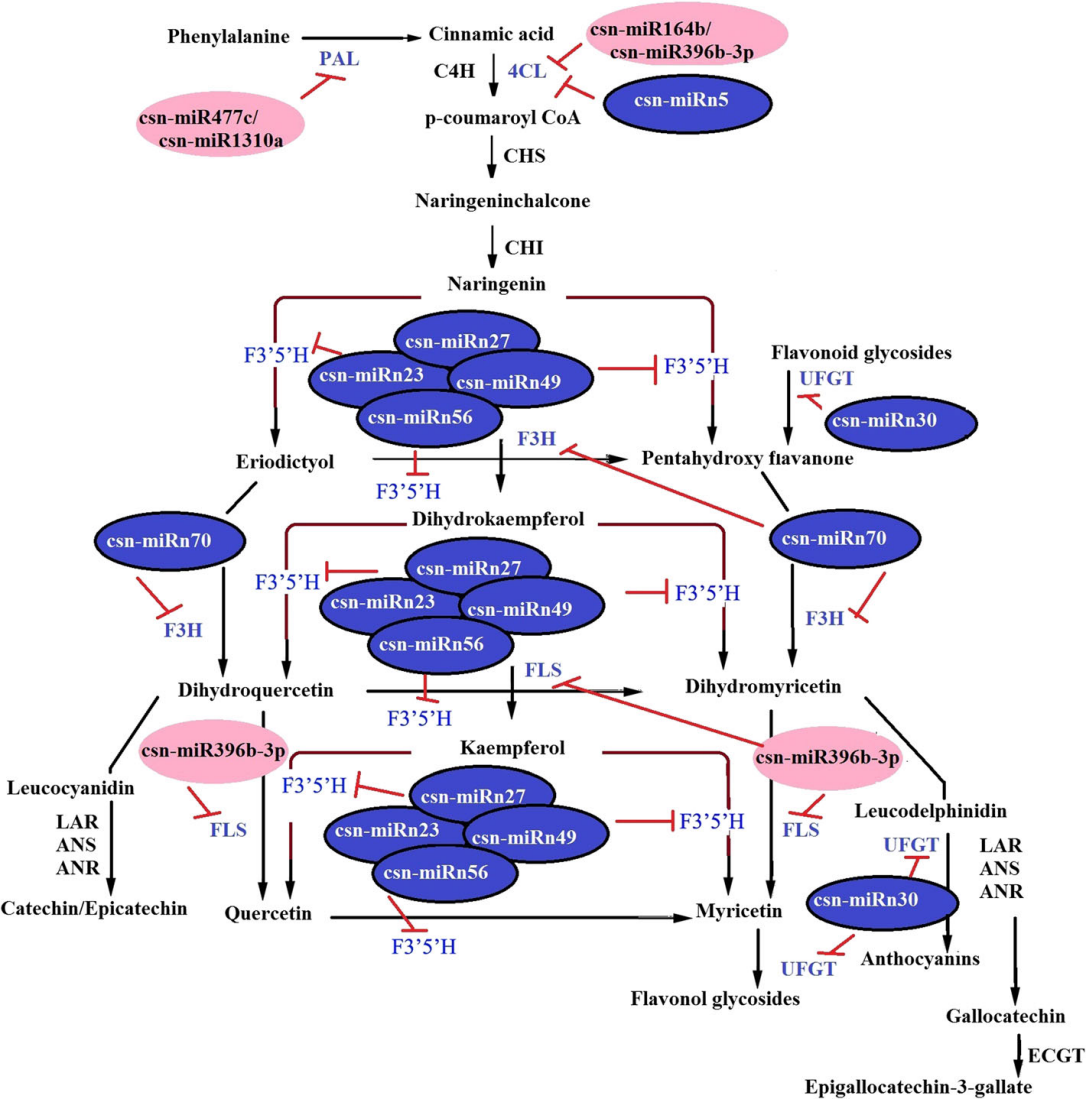
Hypothetical model of csn-miRNA regulation in phenylpropanoid biosynthesis pathway. Pink and blue circles with miRNA names denote conserved and novel miRNAs identified in this study. The putative target transcripts of csn-miRNAs are indicated as abbreviated letters in blue. The miRNA regulations are shown as red inhibition bars. *PAL*, phenylalanine ammonia lyase; *4CL*, 4-coumarate-CoA ligase like 5; *C4H*, 4-coumarate--CoA ligase; *CHS*, chalcone synthase; *CHI*, chalcone isomerase; *F3′H*, flavanone 3-hydroxylase; *F3′5′H*, flavonoid 3′,5′-hydroxylase; *FLS*, flavonol synthase; *LAR*, leucoanthocyanidin reductase; *ANS*, anthocyanidin synthase; *ANR*, anthocyanidin reductase; *UFGT*, UDP-glycose flavonoid glycosyltransferase; *ECGT*, epicatechins 1-O-galloyl-β-D-glucose O-galloyltransferase

KEGG pathway enrichment analysis showed that the target genes of the miRNAs were mainly involved in 16 pathways (*P* ≤ 0.05): phenylpropanoid biosynthesis and nitrogen metabolism were the two most common pathways (Fig. 7 and Additional file 7). In particular, 16% the target genes were involved in phenylpropanoid biosynthesis pathways (Additional file 7). Based on the predicted target gene functions in phenylpropanoid biosynthetic pathway, we propose a pathway panel for polyphenol regulation in tea (Fig. 8). Furthermore, four novel miRNAs (csn-miRn23, csn-miRn27, csn-miRn49, and csn-miRn56) were selected and correlated their expression pattern (Fig. 5) with the pathway panel (Fig. 8). These miRNAs may play an important role in regulating the biosynthesis of phenolic compounds in tea plants.

### Experimental verification of miRNA-guided cleavage of target mRNAs in tea plant

**Fig. 9:**
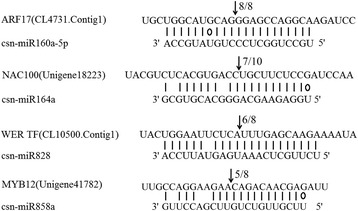
The mRNA cleavage sites identified by 5′ RLM-RACE. Watson-Crick pairing (*vertical dashes*) and G:U wobble pairing (*circles*) are indicated. The arrows indicate the 5′ termini of mRNA fragments isolated from tea plant, as identified by RLM-RACE product, with the frequency of clones shown. The numbers indicate the fraction of cloned PCR products terminating at different positions

To examine the predicted targets of 4 conserved miRNAs, we used 5’RLM-RACE to determine the cleavage sites of miRNA on its target gene. All the 5’RLM-RACE PCR products were analysed on agarose gel, purified, cloned and sequenced. The sequencing results revealed that the cleavage site of ARF17 (CL4731.Contig1), WER transcription facor (CL10500.Contig1) and MYB12 (Unigene41782) lies between 11th and 12th base from 5′ end pairing of csn-miR160a-5p, csn-miR828 and csn-miR858a respectively. NAC100 (Unigene18223) was verified as a target of csn-miR164a. NAC100 can be regulated by cleavage in the binding region between the 10th and 11th base from 5′ end pairing of csn-miR164a (Fig. 9).

### Expression analysis of miRNAs and their target genes in different tissues of tea plant

**Fig. 10:**
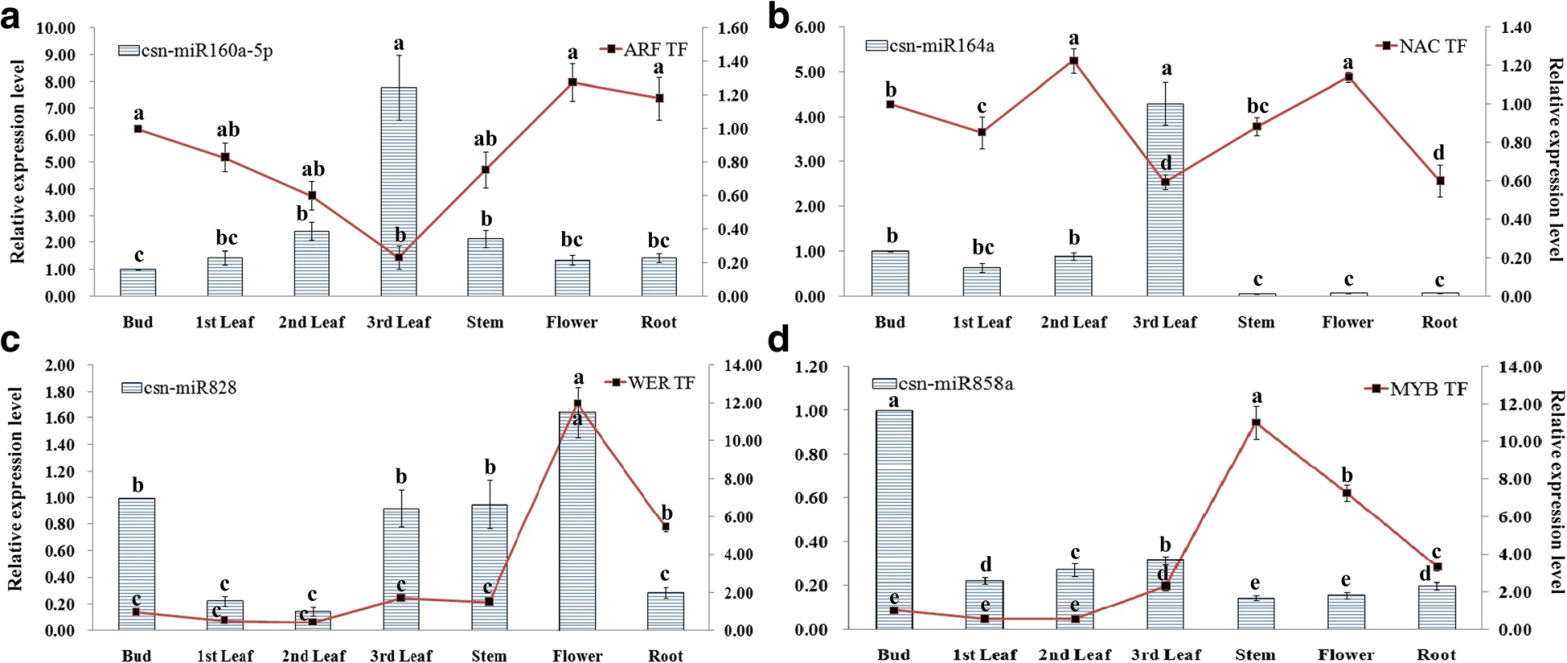
Expression correlation between miRNAs and target genes. **a-d** The bars and lines represent the abundance of miRNAs and their corresponding targets in the seven tea plant tissues. The Y-axis on the left and right indicates the expression levels of the miRNAs and targets, respectively. U6 snRNA and *GADPH* were used as an internal control for miRNAs and targets, respectively. The expression level of the miRNAs and targets in the bud tissue were set as 1.0. Relative expression was calculated using the 2^-△△CT^ method. Data represent the mean ± SD values of three biological replicates. Different letters above the bars represent significant differences at p < 0.05. Means followed by the same letter over the bars are not significantly different at the 0.5% level, according to DMRT analysis

The target transcription factor genes of four conserved miRNAs validated through 5’RLM-RACE, were selected for expression analysis by qRT-PCR. To understand the physiological importance and the regulatory mechanisms of selected miRNAs in tea plant, correlation in the expression pattern of miRNAs and their target genes was determined in different tissues. The expression pattern of csn-miR160a-5p was higher in 3rd leaf, followed by 2nd leaf, stem, 1st leaf, root and flower in comparison to bud; conversely, the opposite trend was observed for the corresponding auxin responsive factor (ARF17). The expression of csn-miR164a and NAC100 also showed negative correlation in 3rd leaf. While a negative correlation between miR858a and MYB12 was observed in stem followed by flower and root, in the case of csn-miR828 and WER transcription factor, the expression of target gene was partially positively correlated with the expression of the miRNA in different tissues (Fig. 10).

## Discussion

In earlier studies, a limited number of miRNAs were identified in tea plants through computational and direct cloning approaches [7–10]. With the development of high-throughput sequencing (HTS) technology in identification of novel and low-abundance miRNAs in plants, many more tea plant miRNAs have been found when investigating the abiotic stress response miRNAs in tea plant. For example, in a study of cold-response miRNAs in tea cultivar YS and BY, 106 conserved miRNAs and 98 potentially novel miRNAs were identified [16]. In a similar study, 295 conserved and 72 potential novel miRNAs were found [17]. In a research of drought tolerance of tea plant, 268 conserved miRNAs and 62 novel miRNAs were detected [23]. However, in these studies, almost all the mature miRNAs and their pre-miRNA secondary structures were predicted based on EST data sets with little information of genomic sequences of tea plant in public database. Most of the pre-miRNA cannot be identified through ESTs due to the lower abundance of primary miRNAs (pri-miRNAs) in miRNA processing and the limited sequencing information of the EST database. To comprehensively identifying miRNAs from plants, the scaffold sequences obtained by genome survey have been used as an important supplementary resource to predict the pre-miRNA secondary structures [13, 14], in addition to ESTs.

In this study, we focused on identifying the miRNAs specifically from one bud and two tender leaves of tea plant and further analyzed their structures using the information of tea plant genome survey sequence scaffolds, which we recently obtained. With a genome survey, we obtained 27.2 × coverage of *C. sinensis* genome sequence followed by assembly to scaffolds. These valuable scaffold data served as important sequence resources in predicting the secondary structures of novel miRNAs by bioinformatics analyses. In total, we identified 175 conserved and 83 novel miRNAs in tea plant, among which pre-miRNA secondary structures of 140 conserved and 69 novel miRNAs were supported by corresponding scaffolds sequences (Additional files 1 and 3). So far, we have not found any miRNA identification studies on tea plant, whose pre-miRNA secondary structures have been confirmed on the base of genome sequences. Thus, our approach to tea plant miRNA identification was much more accurate and reliable than previous studies [16, 17].

Length distribution analysis, as an effective assessment of the composition of small RNAs, showed that 24-nucleotide small RNA sequences were the most dominant in both the total and unique reads, followed by 21- to 23-nucleotide RNA sequences (Fig. 1). This result was highly consistent with previous reports on other tea plant cultivars [16, 17]. Thus, small RNAs that are 24 nucleotides long might play a vital role in *C. sinensis*. Similar patterns in the length distribution of small RNAs were also found in other plant species, such as *Citrus sinensis* [24], *Lycium chinensis* [25], and *Punica granatum* [26], and some monocotyledons [27–30]. However, some studies have shown that small RNAs that are 21 nucleotides long are the most abundant in species such as *Oryza sativa* [31], *Populus euphratica* [32] and *Citrus reticulate* [33]. These variations in the length distribution of small RNAs indicate that the small RNA transcriptomes might be complicated and significantly different between plant species, depending on their regulatory mechanism in different types of plant species.

To explore the evolutionary roles of conserved and non-conserved miRNAs, both types of miRNAs from tea plant were compared against the known miRNAs in five different plant species (*Populus trichocarpa*, *Medicago truncatula*, *Vitis vinifera*, *Oryza sativa* and *Arabidopsis thaliana*). Seven miRNA families (miR1310, miR1427, miR2911, miR5301, miR6149, miR6173 and miR6300) were detected only in *C. sinensis* (Fig. 2), in addition to many miRNAs that are conserved to different extents in plants. We speculated that these seven non-conserved miRNA families might have specific biological functions in tea plant development, which requires further investigations.

Genome information of non-model plant species is important in identifying miRNAs and their pre-miRNA stem-loop structures in plant species such as tea [16, 17]. Before genome sequence information is available, genome survey datasets are valuable in miRNA identification. We successfully established tea plant genome survey datasets by WGS, and used them to identify and analyze the stem-loop secondary structures of pre-miRNAs using the mfold software in this study. Our results showed that the lengths of these pre-miRNA structures varied from 38 to 258 nucleotides in tea plant (Additional files 1 and 3), which may involve in the regulation of miRNA biogenesis through the interaction of their unique structures with miRNA pathway enzymes [21].

Mature miRNAs are produced from the genome-encoded stem loop precursor in plants. The majority of 20- to 25-nucleotide miRNAs are processed from a pre-miRNA (~70-nucleotides long hairpin precursor), which forms a hairpin structure containing mature miRNA in either of its arms [34]. Therefore, these miRNAs (20–25 nucleotides in length) were used to search against miRNA precursors in the reference dataset, including EST and the genome survey sequences in this study. Our results showed that all the identified miRNAs were in their corresponding standard stem-loop hairpin secondary structures (Additional files 1 and 3). Minimum folding free energy (MFE) is a significant characteristic that determines the secondary structure and stability of nucleic acids (DNA and RNA). The predicted miRNA precursors have an average MFEI value of −57.08 kcal/mol, which is higher than that of tRNA (−27.5 kcal/mol) and rRNA (−33 kcal/mol) [35]. Thus, the predicted characteristic and thermodynamically stable secondary structure of the pre-miRNAs in this study was consistent with that reported in previous studies on tea [16, 17].

Microarray detection is widely used to confirm the presence of miRNAs in plants [36, 37]. Here, we designed probes for conserved and novel tea plant miRNAs and developed microarray platform to analyze mature tea miRNA profiles, and detected altogether 93 conserved and 18 novel miRNAs in tea (Additional file 4). The miRNAs from the csn-miR5368 and csn-miR6173 families, which are conserved miRNA families, displayed high expression signals, while low expression signals were observed for conserved miRNA families csn-miR477, csn-miR482-5p, csn-miR858b, csn-miR156, csn-miR395 and csn-miR403. With regard to the novel miRNAs, csn-miRn5 and csn-miRn11-3p showed higher expression signals than the other miRNAs (Additional file 4). A previous study reported that miR477 and miR482 were differentially expressed during drought stress in the root and leaf of bread wheat; moreover, miR156 was reported to be down regulated in response to drought stress in rice [38]. In addition, miR858 was reported to be differentially expressed in different tissues of the tomato, and to negatively regulate anthocyanin biosynthesis under normal growth conditions [39]. In *Spartina alterniflora*, miR395 was reported to be downregulated by salt-induced stress and play important regulatory roles in plant growth and development [40]. Ebrahimi et al. [41] reported that miR403 was differentially expressed in response to abiotic stresses such as drought, heat, salt and cadmium in the sunflower. These observations indicate that miR156, miR395, miR403, miR477 and miR482 are most likely to be implicated in the response to abiotic stresses also in the tea plant. However, further studies on more specific functions of these miRNAs are required.

It is well known that transcription factors play significant roles in plant development, stress responses and secondary metabolism. For example, *SPLs*, a family of transcription factors specific to plants, which were targeted by miR156, were found to be involved in a number of stress response processes, including response to heat stress, salt stress and drought stress, in plants [42, 43]. In this study, some miRNAs, such as those of the miR172 family (csn-miR172a-3p, csn-miR172c and csn-miR172d) and csn-miRn61, were predicted to target ethylene-responsive transcription factors, which may control the biosynthesis of ethylene and regulate the activation of the ethylene pathway [44]. Ethylene is the simplest but one of the most important phytohormones, which participates in major developmental processes, including seed germination, cell elongation, flowering, fruit ripening, organ senescence, abscission, and response to stress [45]. In addition, csn-miR395a-3p was predicted to target *WRKY* transcription factors, which exert an key function in abiotic stress responses in plants [46, 47]. However, other miRNAs like csn-miR828 and csn-miR858 families were predicted to target transcription factor *WER* and *MYB*. The transcription factor *WER* encodes a putative transcription factor of the *MYB* family transcription factor which is the largest family of transcription factors that have significant functions to promote differentiation of non hair cells and involved in various regulatory networks by controlling development, metabolism and responses to biotic and abiotic stresses [48]. In this study, we confirmed through 5’RLM-RACE that csn-miR828 and csn-miR858a were predicted to target *WER* transcription factor and *MYB12* respectively and exhibited a negative correlation in expression profile in different tissues of tea plant.

The plant-specific *NAC* family is mainly associated with the regulation of various processes including flower development, formation of secondary walls and cell division and shoot apical meristem formation [49, 50]. Recently, it was shown that the miR164 family targeted six *NAC* family members in several plant species [51, 52]. In our study, we validated *NAC* transcription factor gene as a target of csn-miR164a through 5’RLM-RACE. We also observed a negative correlation in the expression pattern of csn-miR164a and *NAC100* in 3rd leaf, 2nd leaf and 1st leaf. Csn-miR160a-5p is predicted to target auxin responsive factor (*ARF17*) that have a crucial function in the response to various abiotic stresses in different plant species by fine-tuning plant growth and development [53]. Additionally, our study has also confirmed through 5’RLM-RACE that csn-miR160a-5p targets *ARF17* and has inverse correlation between csn-miR160a-5p and *ARF17,* demonstrated by qRT-PCR. Based on this result, we observed that predicted miRNAs were likely to target various mRNAs encoding transcription factors. These miRNA-TFs interactions not only serve as a basis for elucidating the regulation and function of miRNAs but also play a key role in polyphenol regulatory network by controlling various plant growth and development in tea plant.

Polyphenols are the most abundant secondary metabolites present in the leaves of the tea plant, they account for 18% to 36% of the dry weight of fresh leaves in most tea cultivars [54], even for higher than 36% in some cultivars, for example, *C. sinensis var. assamica* cv. Jianghua. In *Arabidopsis* spp., it has been proven that repression of miR156 activity resulted in the production of high levels of flavonols through miR156-targeted *SPL* genes [55]. In addition, miR828 in *Arabidopsis* spp. was reported to silence the *MYB113* gene, which regulates the biosynthesis of anthocyanins [56]. It has recently been hinted that Cs-miR156 might reduce the expression level of the target gene *SPL* to regulate the dihydroflavonol 4-reductase (*DFR*) gene, which is a key gene in catechin biosynthesis [57]. In this study, we found that miRNAs of the miRNA156 family, including csn-miR156a, b, c, d, e, and h, might target the *SPL4* gene, and that the *MYB308* gene may be a possible target of csn-miR828 (Additional file 6). Thus, miRNAs of the tea plant may exert an important contribution in the biosynthesis of phenolic compounds, which are an important constituent of tea. In particular, the findings in present study indicate the need for further studies on the csn-miR156 family-regulated *SPL4* gene and the csn-miR828-regulated *MBY308* gene, as they may undertake a role in the accumulation of polyphenols in the tea plant.

Catechins are the most abundant important secondary metabolites, as they account for 70% of all polyphenols in tea leaves [58]. They are synthesized mainly via multiple branches of the phenylpropanoid biosynthetic pathway [54, 58]. Flavonoid 3′-hydroxylase (F3′H) and flavonoid 3′,5′-hydroxylase (F3′5′H) are key enzymes concerning in the formation of di- and tri-hydroxylated catechins [59, 60], which are important as indicators of tea quality and biochemical markers in genetic diversity studies [60]. KEGG annotation analysis of the target transcript functions revealed that four conserved miRNAs (the csn-miR477e/csn-miR1310a pair, csn-miR164b, and csn-miR396b-3p) were involved in phenylpropanoid biosynthesis (Additional file 7), and four novel miRNAs (csn-miRn23, csn-miRn27, csn-miRn49 and csn-miRn56) were found to target a single transcript encoding F3′5′H (Additional file 5 and Fig. 8). These results indicate that these miRNAs may execute an important function in the biosynthesis of polyphenol compounds in the tea plant. Further studies on the regulation of F3′5′H genes by csn-miRn23, 27, 49, 56 might enhance our understanding of polyphenol accumulation and regulation in the commercially important tea tissue.

Usually one bud and two leaves of tender tea shoots are used to process commercial teas. In consideration of the important healthy and economic value of leaves of tea plant, the aim of this study is only focus on exploring miRNAs from leaves of tea plant. To comprehensively understanding the miRNA in tea plant as a natural species, we will further speculate miRNAs in other tea plant tissues (mature and old leaves, stem, flowers, seed and root). Although the genome survey was used to support the prediction of miRNAs in present study, this type of data only contained part of the whole genome sequences and may limit the identification of the extensive novel miRNAs. We are expecting to find more miRNAs when the whole genome sequence of tea plant is available.

## Conclusions

In the present study, we identified 175 conserved miRNAs and 83 novel miRNAs from a small RNA library obtained from one bud and two leaves of the tender tea shoot. A number of assembled scaffolds from the genome survey have proven to be valuable for elucidating the potential secondary structures of novel miRNAs without the whole-genome sequence of *C. sinensis* as a reference. In total, 716 target genes of miRNAs were predicted, which were mainly involved in enzyme activity, response to stress, and cellular processes. The highest ranking miRNA target genes might undertake a significant role in the accumulation of polyphenols, which are abundantly found in the tea plant. Furthermore, we verified the potential target transcription factor genes (ARF17, NAC100, WER and MYB12) for selected conserved miRNAs by 5’RLM-RACE, and negative correlations between expression levels of these conserved miRNAs and their targets was validated through qRT-PCR. Therefore, these miRNAs might be involved in various regulatory networks in the tea plant by regulating the expression of ARF17, NAC100, WER TF and MYB12. Our study laid a foundation for further investigation into the molecular mechanisms of the metabolic regulation of tea plants and other closely related species.

## Methods

### Plant material and growth conditions

Shuchazao (*Camellia sinensis* L. var.) was used in the present study, because it was certified as a national variety by the National Crop Variety Approval Committee in 2002 (Accession number: 2,002,008) in China. It can be used to possess both excellent quality and high yield of tea leaves, and has become a very popular variety. Currently, it is grown in six provinces with a total planting area of approximately 20,000 ha. Three-year-old clone cuttings were cultured in pots (30-cm diameter, 35-cm height) and grown under natural daylight conditions at the tea plantation in Anhui Agricultural University, Hefei, China. Various experiments were carried out on “two and a bud” samples (apical bud and the associated leaves up to the second node positions), different tissues (bud, stem, flower, root) and the leaves from the first to fifth positions (position with reference to the apical bud) on the shoots of tea plants. The samples, tissues and leaves were harvested from these cuttings, and immediately frozen in liquid nitrogen and stored at −80 °C till further use.

### Extraction and quantification of total RNA

Total RNA was extracted using the Total RNA Purification kit (NorgenBiotek Corporation, Canada) according to the manufacturer’s protocol. The total RNA quantity and purity were examined using Agilent Technologies 2100 Bioanalyzer (Agilent Technologies, Palo Alto, CA, USA).

### Small RNA library construction and sequencing

Small RNA fragments (16 − 30 nucleotides) were isolated from the 200 μl total RNA pool using polyacrylamide (15%) denaturing gel. After purification, the small RNAs were ligated sequentially to a 5′ RNA adaptor and a 3′ RNA adaptor by *T4* RNA ligase, reverse transcribed to cDNA, and amplified by PCR. Finally, the purified and validated small RNA-derived cDNA library was sequenced by Solexa sequencing technology using an Illumina GAIIX system provided by LC Sciences (Houston, Texas, USA). The generated small RNA library sequences have been deposited in the Gene Expression Omnibus (GEO) database.

### Genome survey using whole-genome shotgun sequencing and de novo assembly

Genome survey-based miRNA identification was performed using the WGS approach [16, 61]. The first leaves were harvested for DNA extraction with the plant genomic DNA extraction kit (Tiangen, Beijing, China) following the manufacturer’s instructions. DNA was randomly sheared by nebulization and end-repaired with *T4* DNA polymerase, and the DNA sequences were selected by size using gel electrophoresis on 1% low-melting-point agarose. Three sequencing libraries of insert size 180 bp, 500 bp and 800 bp were constructed according to the manufacturer’s instructions (Illumina Inc., San Diego, CA, USA). Pair-end sequencing of the constructed libraries was performed on the Illumina HiSeq2000 platform (Illumina, San Diego, CA, USA). Further, the sequences were de novo assembled to prepare a draft scaffold dataset using the SOAP de novo program with a K-mer of 17 [61]. To identify the miRNAs present in tea, the obtained small RNA library sequences were mapped to the draft scaffold assembly sequences of the WGS dataset. Technical support for genome sequencing and initial data analysis was provided by Beijing Genome Institute (BGI), Shenzhen, China.

### Identification of conserved and novel miRNAs

**Fig. 11:**
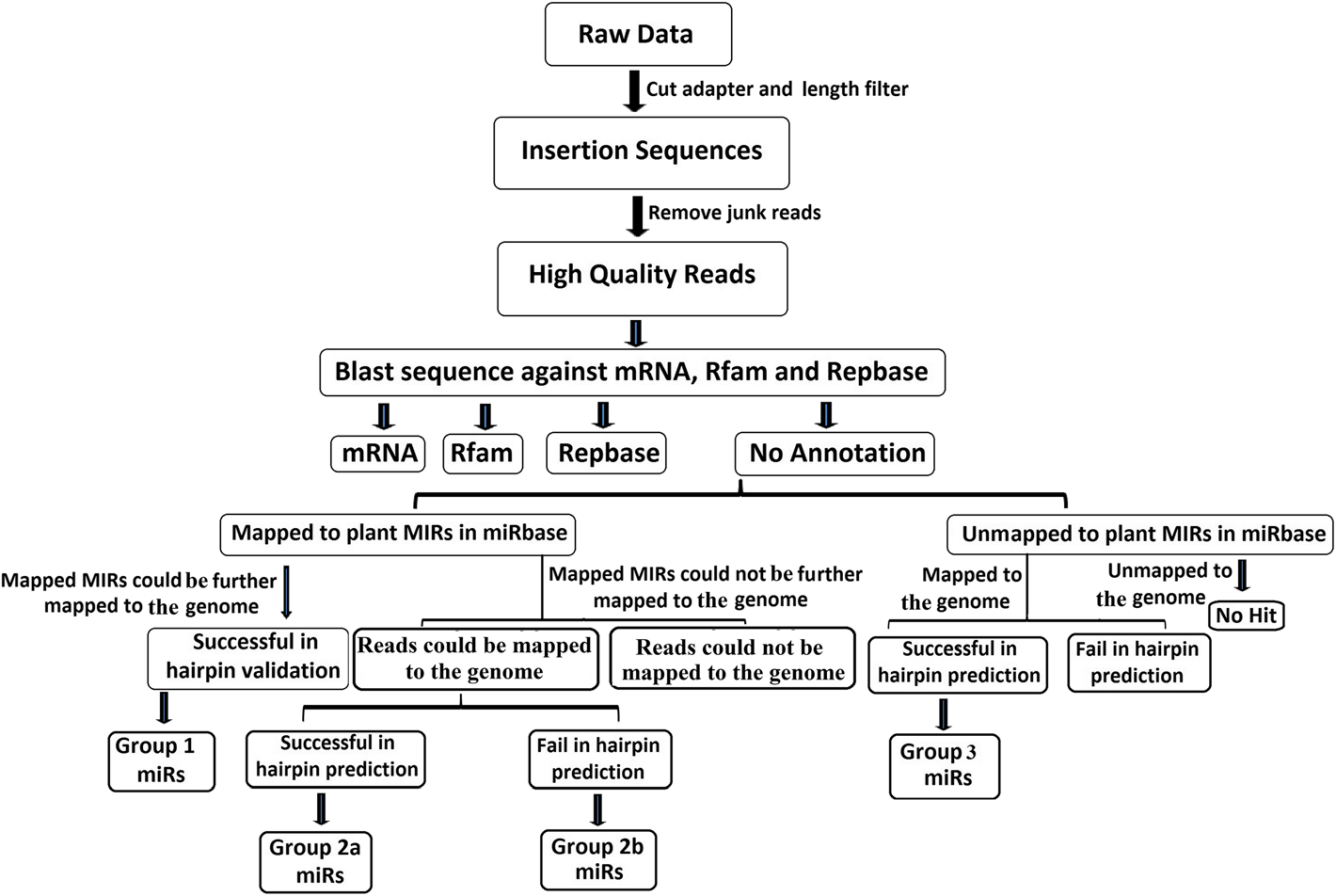
Schematic representation of the miRNA screening procedure used to identify homologues of conserved and novel miRNAs from the tea genome. MIRs and miRs represent pre-miRNAs and mature miRNAs, respectively

The procedure for identification of conserved and novel miRNAs is summarized in Fig. 11. Briefly, to obtain clean reads, the raw reads were filtered using the Illumina pipeline filter (Solexa 0.3), and reads were processed with an in-house program, ACGT101-v4.2-miR (LC Sciences, Houston, Texas, USA) to remove adapter dimers, junk sequences, low–complexity sequences, common RNA families (rRNA, tRNA, snRNA, and snoRNA) and repeats [62, 63]. Subsequently, unique sequences ranging from 17 to 25 nucleotides in size were collected and mapped with mature and pre-miRNAs from other plant species available in the miRBase database (miRBase, Release 21; June 2014) [4]. After the analysis, conserved miRNAs that were mapped to the database sequences were identified and categorized into three groups (1, 2a, and 2b), whereas non-mapped sequences were considered as novel putative miRNAs and grouped into one group (3).

The stem-loop secondary structures of the pre-miRNAs were predicted using the mfold software (version 3.6) (http://unafold.rna.albany.edu/?q=mfold/download-mfold), which was used as a support program for ACGT101-v4.2-miR. The following criteria were used for predicting the pre-miRNA secondary structure: (i) ≥ 12 nucleotides in one bulge of a stem, (ii) ≥ 16 base pairs in the stem region of the predicted hairpin, (iii) length of hairpin (up and down stems + terminal loop) ≥ 50 nucleotides, (iv) length of hairpin loop ≤ 200 nucleotides, (v) ≤ 4 nucleotides in one bulge in mature regions, (vi) biased errors of ≤ 2 nucleotides in one bulge of mature regions, (vii) ≤ 2 biased bulges in mature regions, (viii) errors of ≤ 4 nucleotides in mature regions, (ix) ≥ 12 base pairs in the mature region of a predicted hairpin, (x) ≥ 80% mature regions in a stem, (xi) cut-off of ≤ − 12 kcal/mol of free energy in the secondary structure folding region, (xii) ≥ 0.7 or ≥ 0.4 minimal folding free energy index (MFEI) of secondary hairpin structures based on target gene availability.

### miRNA microarray analysis

miRNA microarray analysis and chip hybridization were performed by LC Sciences (Houston, Texas, USA). Briefly, the extracted small-molecular-weight RNA from one bud and two leaves of tender tea shoots was used for microarray hybridization. RNAs were size-fractionated using the YM-100 Microcon centrifugal filter (Millipore, Bedford, MA, USA). The fractionated small RNAs (<30 nucleotides in length) were extended with a poly-(A) tail at the 3′ end by using poly (A) polymerase and were ligated to an oligonucleotide tag for subsequent fluorescent dye staining. A total of 258 probes were designed to represent the identified miRNAs obtained in HTS, which are completely complementary to the target miRNAs with a chemically modified nucleotide base. The probes were spotted in three replicates onto each chip. Hybridization was performed using 100 μl of hybridization buffer containing 6× SSPE (0.90 M NaCl, 60 mM Na_2_HPO_4_ and 6 mM EDTA at pH 6.8) and 25% formamide at 34 °C in a microcirculation pump (Atactic Technologies, Houston, TX, USA).

Hybridized arrays were analyzed with a laser scanner (GenePix 4000B; Molecular Device, Sunnyvale, CA, USA), and the images were digitized with the Array-Pro image analysis software (Media Cybernetics, Silver Spring, MD, USA).

During the data analysis, the background signal was subtracted and normalized using the LOWESS program. Spot signals that were less than three-fold the background standard deviation (BSD) and had a spot coefficient of variation (CV) greater than 0.5 were removed. To minimize noise and improve accuracy, probes with low abundance (signal value <100) were not included in variance analysis. Signals below the background average (signal value <30) were considered as non-expressing small RNAs.

### Validation of miRNAs using qRT-PCR

The stem-loop qRT-PCR method was used to validate the predicted miRNAs from the small RNA sequencing analysis and determine the transcript levels of the miRNAs [64]. The stem-loop RT primers consisted of 44 conserved and 6 variable nucleotides that are specific to the 3′ end of the miRNA sequences (5′GTC GT A TCC AGT GCA GGG TCC GAG GTA TTC GCA CTG GAT ACG CAN NNN NN3′). Forward primers, in which six nucleotides at the 3′ end of the stem-loop RT primer were complementary to the 3′ end of the miRNA, were designed for each individual miRNA according to Varkonyi-Gasic et al. [65], and 5′-GTG CAG GGT CCG AGG TAT TC-3′ was used as the reverse primer. U6 was used as an internal control. Detailed information regarding the primers used in this study is provided in Additional file 8.

cDNAs were synthesized in a 20 μl solution containing 500 ng of total RNA, 4 μl 5× PrimeScript buffer, 0.5 μl M-MLV reverse transcriptase (Takara, Dalian, China), and 1 μl stem-loop RT primer (1 μM). After pre-denaturation at 65 °C for 5 min, the mixture was incubated on ice for 2 min, and the RT reaction was performed for 30 min at 16 °C. This was followed by 60 cycles at 30 °C for 30 s, 42 °C for 30 s and 50 °C for 1 s, and a final hold at 85 °C for 5 min.

qRT-PCR was conducted on the CFX96 real-time detection system (Bio-Rad, Hercules, USA) using a SYBR Premix Ex TaqTMII kit (Takara, Dalian, China). The final volume of the solution was 25 μl, and it contained 2 μl cDNA, 8.5 μl ddH_2_O, 12.5 μl SYBR Premix Ex TaqTMII, 1 μl specific forward primers (10 μM) and 1 μl universal reverse primers (10 μM). The reaction solution was incubated at 95 °C for 5 min; this was followed by 40 cycles at 95 °C for 5 s and 60 °C for 10 s.

The expression level of randomly selected miRNAs for validation of the predicted miRNAs was calculated using the 2^ΔCt^ method [66]. Ct values were determined automatically by the in-built software, based on the formula ΔCt = Ct_U6_ − Ct_miRNA_. The relative expression of the selected miRNAs in the leaves at different node positions was quantified using the 2^–ΔΔCt^ method and expressed as the fold change relative to the expression in the first leaves (set as 1) [67]. Quantity of selected miRNA accumulation levels in different tissues of tea plant were calculated as relative expression values in comparison to bud tissues using the 2^–ΔΔCt^ method [67]. The amplification efficiency for all the selected miRNAs tested in this study ranged from 95% to 110% was autocalculated from the slope of the standard curve by the CFX manager software. All qRT-PCR analyses were performed in three biological replicates, each of which consisted of three technical replicates.

### Target gene prediction

To predict potential target genes, all miRNAs obtained with HTS were analyzed by Target Finder (https://github.com/carringtonlab/TargetFinder Finder) against the transcriptome sequence data of *C. sinensis*, and deposited in the NCBI Sequencing Read Archive database under accession number SRR1979118. The predicted target genes were evaluated based on complementarity scoring and maximum expectation, according to the method described by Allen et al. [68].

### GO and KEGG analysis

To determine the functions of the target genes and their corresponding metabolic network regulated by miRNAs, functional annotation of the target genes was performed using GO (http://www.geneontology.org/) mapping for molecular functions, biological processes and cellular components. The metabolic pathways were annotated using maps from the KEGG (http://www.genome.jp/kegg/) database [69]. We applied a hyper-geometric distribution statistic to ensure that the target genes were matched with their corresponding biological metabolic pathways, which was indicated by *p*-values less than 0.05 according to Fisher’s exact test.

### Verification of miRNA target genes by 5’RLM-RACE

The cleavage site of predicted miRNA targets were validated through 5’RLM-RACE using FirstChoice RLM-RACE Kit (Invitrogen, Thermo Fisher Scientific) according to the manufacturer’s protocol. Briefly, 10 μg of total RNA was ligated to the 5’RNA adapter using T4 RNA ligase and reverse transcribed to cDNA. Further, amplification of cleaved products of miRNA target genes was performed using target gene specific reverse primers and RNA adapter specific forward primers (Additional file 8). The final RLM-RACE products were analysed on agarose gel, purified using the DNA gel extraction kit (Corning Life Sciences, Suzhou, China) according to the manufacturer’s instruction, directly cloned into a pEASY-T1 vector (TransGen Biotech, Beijing, China), transformed into *Escherischia coli* Trans1-T1 competent cells (TransGen Biotech) and sequenced. The sequencing results were analysed to map the cleavage sites. The primers used to amplify cleavage products of tea miRNA target genes through 5’RLM-RACE are listed in Additional file 8.

### Real-time PCR analysis for expression of selected miRNA target genes

The qRT-PCR was carried out to examine the expression pattern of miRNA target genes. Total RNA was isolated from various tissues of tea plant (Bud, 1st leaf, 2nd leaf, 3rd leaf, stem, flower and root). 500 ng of total RNA from samples was reverse transcribed to cDNA using PrimeScript™ RT Master Mix (Takara, Dalian, China) as per manufacturer’s instructions. The first-strand cDNA was used as a template for qRT-PCR with target gene specific primers and SYBR Premix Ex Taq™ II Master Mix (Takara, Dalian, China) in CFX96 connect real-time detection system (Bio-Rad, Hercules, USA). The expression level of miRNA target genes was determined by calculating fold change in selected tissues relative to bud tissue using the 2^–ΔΔCt^ method. *GADPH* was used as an reference internal control. All qRT-PCR analyses were performed in three biological replicates, each of which consisted of three technical replicates. Primers used in qRT-PCR are provided in Additional file 8.

### High-performance liquid chromatography (HPLC) analysis of catechin content

To examine the catechins present in leaf tissues, 50 g of fresh tea leaf tissue was extracted and dissolved in ethanol. The ethanol solution was evaporated, dissolved in hot water and extracted three times with ethyl acetate. The organic phase was concentrated, dried and re-dissolved in 1 mL of methanol. The catechins present in the methanolic solution were analyzed by HPLC. All samples were filtered through a 0.22 μm filter membrane and separated on a Phenomenex Synergi 4u Fusion-RP80 column (250 × 4.6 mm) with detection set at 280 nm using an HPLC-UV detector (Waters 2478, Waters Instruments) according to Liu et al. [70].

### Statistical analysis

All expression data obtained in this study were presented as the mean ± standard deviation values. The expression profile of the miRNAs identified by qRT-PCR was subjected to Duncan’s multiple range test (DMRT) using the DPS software (http://www.chinadps.net) [71].

## Additional files

Additional file 1: List of conserved csn-miRNAs identified by HTS in *Camellia sinensis*. (XLS 127 kb)

Additional file 2: List of csn-miRNAs mapped with tea EST and the assembled WGS dataset. (XLS 2879 kb)

Additional file 3: List of novel csn-miRNAs identified by HTS in *Camellia sinensis*. (XLS 77 kb)

Additional file 4: Microarray analysis of the identified conserved and novel csn-miRNAs. (XLS 35 kb)

Additional file 5: List of putative target transcripts of the identified conserved and novel csn-miRNAs. (XLS 131 kb)

Additional file 6: GO analysis of the putative target transcripts of the identified csn-miRNAs. (XLS 721 kb)

Additional file 7: KEGG pathway analysis of the putative target transcripts of the identified csn-miRNAs. (XLS 60 kb)

Additional file 8: Details of primers used in qRT-PCR and 5’RLM-RACE. (XLS 26 kb)

## Abbreviations

5’RLM-RACE: RNA ligase-mediated rapid-amplification of 5’cDNA ends
BSD: Background standard deviation
CV: Coefficient of variation
ESTs: Expressed sequence tags
F3′5′H: Flavonoid 3′,5′-hydroxylase
F3′H: Flavonoid 3′-hydroxylase
Gbp: Giga base pairs
GO: Gene ontology
HTS: High-throughput sequencing
KEGG: Kyoto encyclopedia of genes and genomes
MFE: Minimum folding free energy
miRNAs: MicroRNAs
WGS: Whole genome shotgun
